# Gene expression profiling of cholangiocarcinoma-derived fibroblast reveals alterations related to tumor progression and indicates periostin as a poor prognostic marker

**DOI:** 10.1186/1476-4598-9-13

**Published:** 2010-01-24

**Authors:** Kusumawadee Utispan, Peti Thuwajit, Yoshimitsu Abiko, Komgrid Charngkaew, Anucha Paupairoj, Siri Chau-in, Chanitra Thuwajit

**Affiliations:** 1 Department of Biochemistry, Faculty of Medicine, Khon Kaen University, 123 Mitraparb Road, Muang, Khon Kaen 40002, Thailand; 2 Department of Immunology, Faculty of Medicine Siriraj Hospital, Mahidol University, 2 Prannok Road, Bangkok Noi, Bangkok 10700, Thailand; 3 Department of Biochemistry and Molecular Biology, Nihon University School of Dentistry at Matsudo, Matsudo, Chiba 2718587, Japan; 4 Department of Pathology, Faculty of Medicine Siriraj Hospital, Mahidol University, Thailand; 5 Department of Pathology, Faculty of Medicine, Khon Kaen University, Thailand; 6 Department of Surgery, Faculty of Medicine, Khon Kaen University, Thailand; 7 Liver Fluke and Cholangiocarcinoma Research Center, Faculty of Medicine, Khon Kaen University, Thailand

## Abstract

**Background:**

Fibroblasts play important roles in several cancers. It was hypothesized that cholangiocarcinoma (CCA)-associated fibroblasts (Cfs) differ from non-tumorigenic liver fibroblasts (Lfs) in their gene expression profiles resulting in the capability to promote cancer. Periostin (PN) is a multi-functional protein and has emerged as a promising marker for tumor progression. The role of PN in CCA, however, has not yet been explored.

**Results:**

In this study, the gene expression profile of Cfs in comparison to Lfs was performed using oligonucleotide microarrays. The common- and unique-expressed genes in Cfs and the promising roles in cancer promotion and progression were determined. PN was markedly over-expressed in Cfs confirmed by real time RT-PCR and western blot analysis. Immunohistochemistry examination of a number of patients with intrahepatic CCA showed the expression of PN solely in stromal fibroblasts, but was expressed neither in cancer cells nor immune cells. Low to no expression of PN was observed in tissues of benign liver disease and hepatocellular carcinoma. CCA patients with high levels of PN had significantly shorter survival time than those with low levels (*P *= 0.026). Multivariate analysis revealed high levels of PN (*P *= 0.045) and presence of lymph node metastasis (*P *= 0.002) as independent poor prognostic factors. The *in vitro *study revealed that recombinant PN induced CCA cell proliferation and invasion. Interestingly, interference RNA against integrin α_5 _significantly reduced the cellular response to PN-stimulated proliferation and invasion.

**Conclusion:**

The gene expression profile of fibroblasts in CCA is apparently explored for the first time and has determined the genes involving in induction of this cancer progression. High PN can be used to distinguish CCA from other related liver diseases and is proposed as a prognostic factor of poor survival. Regulation of fibroblast-derived PN in CCA proliferation and invasion may be considered as an alternative therapeutic approach.

## Background

Cholangiocarcinoma (CCA) originates from biliary epithelial cells and is a unique cancer in northeastern Thailand where the prevalence of a liver fluke, *Opisthorchis viverrini *infection is higher than elsewhere in the country. A recent study showed a strong positive correlation of CCA incidence and the prevalence of *O. viverrini *infection [1]. In other countries, CCA has been shown to correlate with *Clonorchis sinesis *[2,3], and chronic biliary diseases [4]. Even though CCA is caused from the different etiologies, it is well recognized to contain an abundant fibrous stroma that is mainly composed of α-smooth muscle actin (SMA) positive fibroblasts [5,6]. In addition, the degree of α-*SMA *expression has been shown to correlate with the survival of patients, in part, via the ability of these cancer-associated fibroblasts to induce proliferation of bile duct epithelial and cancer cells [6].

The ability of stromal fibroblasts to generate a favorable microenvironment for cancer cells leading to cancer development, invasion and metastasis has been summarized [7,8]. Mitotic substances have been produced from stromal fibroblasts to promote tumor growth in many cancers [9,10]. In addition, some matrix metalloproteinases which are often mentioned as proteolytic substances of the extracellular matrix (ECM) and have been reported to show increased production from cancer stromal fibroblasts. These proteolytic substances appear to help to promote cancer cell invasion and metastasis [11]. Specifically, in CCA, stromal-derived factor 1 (SDF-1) has been secreted from stromal fibroblasts into the microenvironment in which it was located at the edge of cancer masses and was proposed to play important role in induction of CCA cell invasion and metastasis [12].

To understand the roles of fibroblasts in carcinogenesis, cancer promotion and progression, gene profiling of cancer fibroblasts have been studied in many cancers [13-15]. Stromal cancer fibroblasts from breast cancer with invasion were compared with the expression profiles of fibroblasts in benign breast disorders. *HYL *(Csk-homologous kinase CHK) involving in regulation of Src kinase, *GM CSF-1 (*granulocyte monocyte colony stimulating factor-1) and *osteopontin *were up-regulated which may result in induction of tumor growth and metastasis [13]. Among genes encoded for secreted proteins over-expressed in fibroblasts of human basal cell carcinoma [14], genes including *CTSK *(cathepsin K), *SFRP2 *(secreted frizzled-related protein 2), *PDGFRL *(platelet-derived growth factor receptor-like protein), and *DCN *(decorin) were shown to be up-regulated in non-epithelial cells of breast cancer [16]. In contrast, these genes could not be detected in fibroblasts isolated from cancers of pancreas [17], and liver metastases of colon cancer [18]. Taken together, it is possible to say that differential gene expression profile of cancer fibroblasts is partly similar but actually unique for each cancer type. This supports the importance of specific recognition of the concerted performance between fibroblasts and epithelial cells in carcinogenesis and progression in different organs of origin. So it is of great value to investigate the specific gene expression profile of the CCA-derived fibroblasts to help us better understand the molecular mechanisms that fibroblasts use to promote cancer.

Periostin (PN) is a secreted protein which was first identified in bone and implicated in regulating adhesion and differentiation of osteoblasts. The cancer biology role of PN has been investigated in a wide range of cancers including cell proliferation [19,20], migration [21], invasion/metastasis [22,23], and angiogenesis [23,24]. When not regarding the specific sources, either from cancer cells or fibroblasts, secreted PN has been reported to induce tumorigenic properties of epithelial cells via the activation of integrins (ITGs) receptors [21].

Even though substantial evidence has shown that cancer-associated fibroblasts are involved in tumor promotion and with the evidence that fibroblasts in CCA induce more aggressive tumorigenic properties of cancer cells [6], the role of CCA-derived fibroblasts in this cancer is yet to be determined. In the present study, fibroblasts isolated from CCA tissues or CCA-associated fibroblasts (Cfs) which were already characterized by the present group [6], were explored. The genome wide expressions of these Cfs were determined and compared to non-tumorigenic liver fibroblasts (Lfs). The altered expression of genes focusing on the impact of soluble products from Cfs on the promotion and progression of CCA was investigated. Interestingly, PN, which has never been reported in CCA was found at a high level whereas no-to-low PN was detected in non-tumor liver tissues and cancer of hepatocytes. The overexpression of PN in CCA tissues was detected solely in fibroblasts and associated with poor prognosis and short survival of the patients. The effect of PN to induce cell proliferation and invasion has been examined.

## Results

### Gene expression analysis of Cf and validation by real time RT-PCR

**Figure 1:**
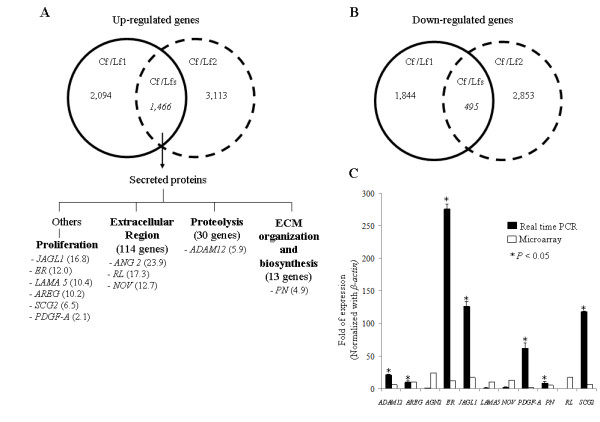
**Genome wide expression analysis of Cf and Lfs and gene validation by real time PCR**. A Vane diagram showed common up-regulated genes (A) and common down-regulated genes (B) in Cf (Cf/Lfs). In this study, eleven genes encoded secreted proteins involved in induction of epithelial cell tumorigenesis including proliferation, invasion, metastasis and angiogenesis were selected from 4 main different groups of biological functions (A). The numbers in the parentheses represent folds of gene expression level of Cf over those in Lfs. The results show the comparison of gene expression levels measured by real time PCR and oligonucleotide microarray (C). Folds of expression represent fold changes of gene expression level in Cfs as compared to that in Lfs.

To reduce the genetic background of different patients, the gene expression profile of Cfs was compared to those of two Lfs namely Lf1 and Lf2. Lf1 was isolated from non-tumorigenic liver tissues of hepatectomized liver from the CCA patient who Cfs were originated from. The Lf2 was isolated from the other CCA patient. Genes with differential expressed levels in Cf compared to Lf1 were 3,560 for 2-fold or more up-regulation and 2,339 for 0.5-fold or less down regulation (Fig 1A and 1B). The comparison of the Cf to Lf2 was 4,579 and 3,348 for up- and down-regulation. The common differential genes which are genes altered in their expressions in Cfs when compared to both Lf1 and Lf2 (Cf/Lfs), were 1,466 for up-regulation and 495 for down-regulation. Arylacetamide deacetylase (*DAC*), procollagen C endopeptidase enhancer 2 (*PCPE2*), serpin peptidase inhibitor (*PAI*) and S100 calcium binding protein A4 (*S100A4*) were predominantly over-expressed at high levels in Cfs whereas bone morphogenic protein 2 (*BMP2*), matrix-remodeling associated 5 (*DKFZp564I1922*), bradykinin receptor B1 (*BRADYB1*), response gene to complement 32 (*RGC32*) and interleukin 24 (*IL-24*) were down-regulated with a high array intensity (Table 1).

**Table 1:** List of top 20 common up-regulated genes and top 20 common down-regulated genes.

Gene	Abbreviation	Intensity	Mismatch	Ratio
Common up-regulated genes		of Cf	binding	Cf/Lfs
arylacetamide deacetylase (esterase)	*DAC*	115.92	P	956.45
sparc/osteonectin (testican 3)	*SPOCK3*	16.70	P	669.24
neuropeptide Y receptor Y1	*NPYR*	20.86	P	416.71
collagen, type XIV, alpha 1 (undulin)	*COL14A1*	47.74	P	245.03
growth associated protein 43	*B-50*	23.52	P	232.10
procollagen C-endopeptidase enhancer 2	*PCPE2*	117.81	P	224.83
sorbin and SH3 domain containing 2	*SORB2*	10.65	P	192.25
myozenin 2	*MYOZ2*	6.53	P	139.50
serpin peptidase inhibitor, clade B (ovalbumin), member 2 transcript variant 2	*PAI2*	260.13	P	133.32
doublecortin-like kinase 1	*DCLK*	19.11	P	112.57
formyl peptide receptor-like 2	*FPRL2*	3.24	P	111.02
contactin associated protein-like 3	*CASPR3*	5.09	P	106.75
integrin, beta-like 1 (with EGF-like repeat domains)	*ITGBL1*	13.35	P	82.92
collagen, type IV, alpha 6	*COL4A6*	76.71	P	77.06
myc target 1	*MYCT1*	28.15	P	73.88
S100 calcium binding protein A4	*S100A4*	103.88	P	72.30
phosphodiesterase 1A, calmodulin-dependent	*HSPDEA1*	12.50	P	71.51
neurofilament, light polypeptide 68 kDa	*NEFL*	5.71	P	69.33
ADAMTS-like 1	*ADAMTSR1*	3.24	P	68.55
early B-cell factor 1	*EBF*	4.57	P	60.80

ST6 (alpha-N-acetyl-neuraminyl-2,3-beta-galactosyl-1,3)-N-acetylgalactosaminide alpha-2,6-sialyltransferase 5	*SIAT7E*	0.02	P	3943.41
fibrillin 2 (congenital contractural arachnodactyly)	*FBN2*	0.05	P	1035.17
fibroblast growth factor receptor 2	*FGFR2*	0.04	P	684.03
pregnancy specific beta-1-glycoprotein 5	*PSG*	0.07	P	349.77
Sal-like 1 (Drosophila)	*SALL1*	0.03	P	312.29
membrane metallo-endopeptidase	*MME*	0.05	P	274.25
odz, odd Oz/ten-m homolog 2 (Drosophila)	*TEN-M2*	0.93	P	134.10
R-spondin 3 homolog (*Xenopus laevis*)	*RSPO3*	0.4	P	70.16
bone morphogenetic protein 2	*BMP2A*	1.19	P	68.96
neuroligin 4, Y-linked	*NLGN4Y*	0.09	P	68.62
matrix-remodelling associated 5	*DKFZp564I1922*	1.31	P	57.89
collagen, type IV, alpha 4	*COL4A4*	0.67	P	49.71
bradykinin receptor B1	*BRADYB1*	1.61	P	48.96
microfibrillar-associated protein 4	*MFAP4*	0.3	P	32.40
matrix metallopeptidase 3 (stromelysin 1, progelatinase)	*MMP-3*	0.85	P	30.01
chromosome 13 open reading frame 15	*RGC32*	1.69	P	28.40
fibroblast growth factor 13	*FGF13*	0.38	P	28.15
ephrin receptor A5	*EPHA5*	0.40	P	25.68
interleukin 24	*IL-24*	1.68	P	25.27

Only those having transcripts, not EST or clones in cDNA library are listed.

P = presence to detectable intensity

Most of common differentially expressed genes in Cfs play roles in controlling cellular metabolism (Table 2 and Table 3). The up-regulated genes encoded for secreted proteins were mostly classified in groups of extracellular region, proteolysis, and ECM organization/biosynthesis which took up to 11% of total genes (Table 2). Among these genes in addition to the secreted protein encoding genes that act in cell proliferation and motility, 11 genes having several tumorigenic functions were selected for further exploration including a disintegrin and matrix metalloproteinase 12 (*ADAM12*), amphiregulin (*AREG*), angiopoietin (*AGN2*), epiregulin (*ER*), jagged1 (*JAGL1*), laminin alpha 5 (*LAMA5*), nephroblastoma over expressed (*NOV*), platelet-derived growth factor-α (*PDGF-A*), periostin (*PN*), reelin (*RL*), and secretogranin 2 (*SCG2*) (Fig 1A).

**Table 2:** Gene ontology of common up-regulated genes. Only genes in the top-five ranking of each group are shown.

Gene ontology (%)	**Accession no**.	Description	Ratio
Cellular metabolism (23.7%)	NM_000909	neuropeptide Y receptor Y1	416.71
	NM_004734	doublecortin-like kinase 1	112.57
	NM_002961	S100 calcium binding protein A4	72.30
	AF208502	early B-cell factor 1	60.80
	AW004016	ST6 beta-galactosamide alpha-2,6 sialyltransferase 2	59.04

Protein binding (20.8%)	BF449063	collagen, type XIV, alpha 1 (undulin)	245.03
	NM_002045	growth associated protein 43	232.10
	AI659533	sorbin and SH3 domain containing 2	192.25
	BF939176	myozenin 2	139.5
	AF333769	contactin associated protein-like 3	106.75

Signal transduction (10.9%)	AW026543	formyl peptide receptor-like 2	111.02
	NM_004791	integrin, beta-like 1	82.92
	NM_005019	phosphodiesterase 1A	71.51
	AF159570	regulator of G-protein signalling 5	49.64
	W67461	angiopoietin-like 1	49.16

Extracellular region (7.8%)	NM_001086	arylacetamide deacetylase (esterase)	956.45
	AI808090	Sparc/osteonectin, cwcv and kazal-like domains proteoglycan (testican) 3	669.24
	NM_013363	procollagen C-endopeptidase enhancer 2	224.83
	AI889941	collagen, type IV, alpha 6	77.06
	NM_052866	a disintegrin and metalloproteinase with thrombospondin motif-like 1	68.55

Transcription factor (7.5%)	AF332197	sine oculis homeobox homolog 2	44.20
	AI681917	iroquois homeobox protein 3	35.34
	NM_020639	receptor-interacting serine-threonine kinase 4	29.41
	AK023792	PBX/knotted 1 homeobox 2	29.40
	AF208967	paternally expressed 3	26.74

Protein modification (5.5%)	AW975934	Titin	32.01
	NM_020639	receptor-interacting serine-threonine kinase 4	29.41
	NM_000222	v-kit Hardy-Zuckerman 4 feline sarcoma viral oncogene homolog	25.21
	BF446673	hemicentin 1	18.96
	NM_002848	protein tyrosine phosphatase, receptor type, O	14.58

Receptor (4.8%)	BF941499	G protein-coupled receptor 116	35.62
	L35594	ectonucleotide pyrophosphatase/phosphodiesterase 2	32.11
	NM_002820	parathyroid hormone-like hormone	30.44
	U61276	jagged 1 (Alagille syndrome)	19.60
	AK022548	integrin, alpha 7	15.62

Cell differentiation (4.3%)	AA343027	Sema domain, immunoglobulin domain (Ig), short basic domain, secreted, (semaphorin) 3D	67.06
	NM_000216	Kallmann syndrome 1 sequence	27.91
	AL560266	Fc receptor-like A	19.61
	AA127691	neuropilin 2	19.35
	NM_002506	nerve growth factor, beta polypeptide	19.11

Cell adhesion (3.5%)	NM_006727	cadherin 10, type 2 (T2-cadherin)	55.43
	NM_000072	CD36 molecule (thrombospondin receptor)	40.71
	AL573851	endothelial cell adhesion molecule	22.52
	N69091	protocadherin 17	22.32
	AA489646	protocadherin beta 13	19.36

Cell cycle (2.4%)	NM_003914	cyclin A1	34.8
	NM_015714	G0/G1switch 2	26.62
	NM_001759	cyclin D2	16.45
	NM_001992	coagulation factor II (thrombin) receptor	13.90
	AK024082	Tousled-like kinase 2	11.09

Cell motility (2.2%)	NM_005045	reelin	17.30
	NM_003062	slit homolog 3 (Drosophila)	5.12
	M21121	chemokine (C-C motif) ligand 5	5.06
	NM_014795	zinc finger E-box binding homeobox 2	4.85
	D45864	protein kinase, cGMP-dependent, type I	4.74

Proteolysis (2.0%)	NM_001870	carboxypeptidase A3 (mast cell)	20.01
	NM_024539	ring finger protein 128	12.68
	AL574912	protease, serine, 35	11.59
	NM_001873	carboxypeptidase E	9.45
	NM_000892	kallikrein B, plasma (Fletcher factor) 1	6.40

Cell proliferation (2.0%)	U77914	jagged 1 (Alagille syndrome)	16.76
	NM_004624	vasoactive intestinal peptide receptor 1	13.07
	BF514079	Kruppel-like factor 4 (gut)	12.89
	NM_001432	epiregulin	11.98
	BC003355	laminin, alpha 5	10.36

Apoptosis (1.6%)	NM_002575	serpin peptidase inhibitor	133.32
	NM_000557	growth differentiation factor 5	14.79
	NM_003728	unc-5 homolog C (C. elegans)	9.60
	BF432648	tumor necrosis factor receptor superfamily	8.73
	NM_003551	non-metastatic cells 5, protein expressed in (nucleoside-diphosphate kinase)	6.80

ECM organization and biosynthesis (0.9%)	BC001186	protocadherin beta 5	15.90
	M25813	tenascin XB	12.46
	NM_002380	matrilin 2	4.89
	AY140646	periostin, osteoblast specific factor 2	4.89
	NM_004612	transforming growth factor, beta receptor I (activin A receptor type II-like kinase, 53 kDa	4.56

**Table 3:** Gene ontology of common down-regulated genes.

Gene ontology (%)	**Accession no**.	Description	Ratio
Cellular metabolism (31.6%)	NM_030965	ST6 (alpha-N-acetyl-neuraminyl-2,3-beta-galactosyl-1,3)-N-acetylgalactosaminide alpha-2,6-sialyltransferase 5	3943.41
	NM_022969	fibroblast growth factor receptor 2	684.03
	AU152837	Sal-like 1 (Drosophila)	312.29
	NM_007287	membrane metallo-endopeptidase	73.38
	BF589322	R-spondin 3 homolog (Xenopus laevis)	70.16

Signal transduction (17.8%)	NM_001200	bone morphogenetic protein 2	68.96
	NM_000710	bradykinin receptor B1	48.96
	R72286	microfibrillar-associated protein 4	32.40
	NM_004114	fibroblast growth factor 13	28.15
	BE218107	EPH receptor A5	25.68

Transcription factor (11.5%)	AJ277914	LIM homeobox 9	25.01
	NM_001452	forkhead box F2	18.08
	AA705845	transducin-like enhancer of split 4 (E(sp1) homolog, Drosophila)	17.16
	BG261252	ecotropic viral integration site 1	11.58
	NM_020327	activin A receptor, type IB	9.32

Protein modification (7.9%)	AF245505	matrix-remodelling associated 5	57.89
	AA725644	ubiquitin specific peptidase 42	18.73
	NM_001982	v-erb-b2 erythroblastic leukemia viral oncogene homolog 3 (avian)	11.34
	AV727260	protein tyrosine phosphatase, receptor type, D	10.93
	NM_002570	proprotein convertase subtilisin/kexin type 6	9.83

Cell differentiation (6.2%)	NM_000641	interleukin 11	17.51
	BC006454	growth arrest-specific 7	15.20
	M69148	midkine (neurite growth-promoting factor 2)	14.03

	NM_003991	endothelin receptor type B	10.85
	AI758962	EPH receptor A4	7.15

Cell adhesion (5.9%)	NM_001999	fibrillin 2	1035.17
	NM_014893	neuroligin 4, Y-linked	68.62
	AI694562	collagen, type IV, alpha 3	22.21
	NM_005864	embryonal Fyn-associated substrate	8.44
	AU146651	collagen, type XII, alpha 1	4.89

Cell cycle (5.9%)	NM_014059	chromosome 13 open reading frame 15	28.40
	M19701	retinoblastoma 1 (including osteosarcoma)	4.93
	NM_002009	fibroblast growth factor 7	4.44
	NM_014703	Vpr (HIV-1) binding protein	3.92
	AI983033	DEAD/H box polypeptide 12	3.91

Cell motility (4.7%)	NM_002784	pregnancy specific beta-1-glycoprotein 9	69.22
	X99268	twist homolog 1	5.45
	NM_015180	spectrin repeat containing, nuclear envelope 2	3.81
	AI990816	laminin, alpha 1	3.59
	N90777	neuropilin 2	3.57

Cell proliferation (4.5%)	NM_016931	NADPH oxidase 4	5.79
	AF064826	glypican 4	4.31
	NM_004525	low density lipoprotein-related protein 2	3.85
	NM_001963	epidermal growth factor (beta-urogastrone)	3.70
	AF064103	CDC14 cell division cycle 14 homolog A	3.67

Apoptosis (4.0%)	NM_006850	interleukin 24	25.27
	NM_002135	nuclear receptor subfamily 4, group A, member 1	5.48
	NM_003823	tumor necrosis factor receptor superfamily	4.59
	AJ301610	glutamate receptor, ionotropic, kainate 2	3.92
	NM_005809	peroxiredoxin 2	3.90

Only genes in the top-five ranking of each group are shown.

The up-regulated levels of these genes in Cfs were verified by relative quantification using real time RT-PCR. In concordance with microarray data, real time RT-PCR results revealed that *ADAM12*, *AREG*, *ER*, *JAGL1*, *PDGF-A*, *PN *and *SCG2 *had significant up-regulations in Cfs compared to Lfs, but that of *NOV *was not statistically significantly increased (Fig 1C). *ANG2*, *LAMA5*, and *RL*, however, showed the opposite direction to the microarray results.

### Detection of PN expression in Cf and CCA tissues

**Figure 2:**
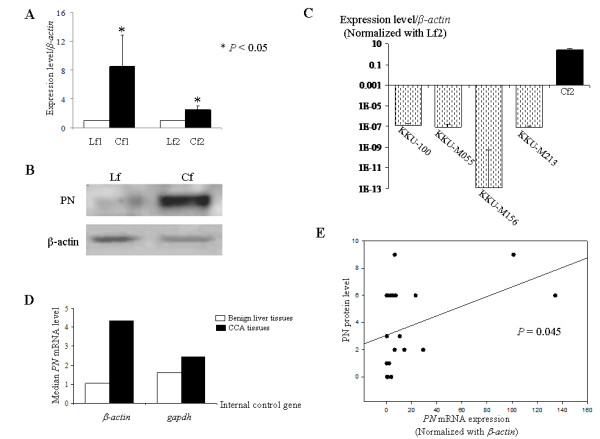
**PN expressions in Cfs, CCA cell lines and CCA tissues**. *PN *expression in Cfs measured by real time RT-PCR (A) and western blot analysis (B) using different biological preparations of Cfs and Lfs from those used in microarray. The expression of *PN *in CCA cell lines and fibroblasts extracted from CCA tissues is graphically depicted (C). Results are expressed as mean ± SD of three independent experiments. Means of *PN *mRNA expression levels were measured in 20 CCA cases and compared to 5 cases of benign liver diseases using both *β-actin *and *gapdh *as internal controls (D). The positive correlation of mRNA and protein levels of PN in CCA tissues is shown with statistical significance by Spearman correlation analysis (E).

Using different biological preparation lots of Cfs from those used in microarray analysis, both real time RT-PCR and western blot analysis confirmed that Cfs had higher expressions of *PN *than Lfs with statistical significance (Fig 2A and 2B). The expression of *PN *in KKU-100, KKU-M055, KKU-M156 and KKU-M213 CCA cell lines was detected at a very low level compared to the high expression in Cfs (Fig 2C).

To check whether the increased expression of *PN *mRNA can be found in CCA tissues, real time PCR was performed using total RNA extracted from pieces of CCA mass. Using *β-actin *and *gapdh *as the internal controls, the results showed the median of *PN *mRNA expression was higher in CCA tissues (4.347 and 2.449 using *β-actin *and *gapdh *respectively) than in benign liver tissues (1.064 and 1.625, respectively) (Fig 2D). This increased up-regulation was not statistically significant. In addition, to achieve the aim to use a rapid method such as real time PCR in place of immunohistochemical detection of PN in CCA tissues, the *PN *mRNA level was related to the intensity of PN immunoreactivity detected by immunohistochemistry. The results indicated the positive correlation of *PN *mRNA level and the encoding protein found in CCA tissues with statistical significance (*P *= 0.045) (Fig 2E).

### Expression of PN in CCA tissues and clinicopathological relevance

**Figure 3:**
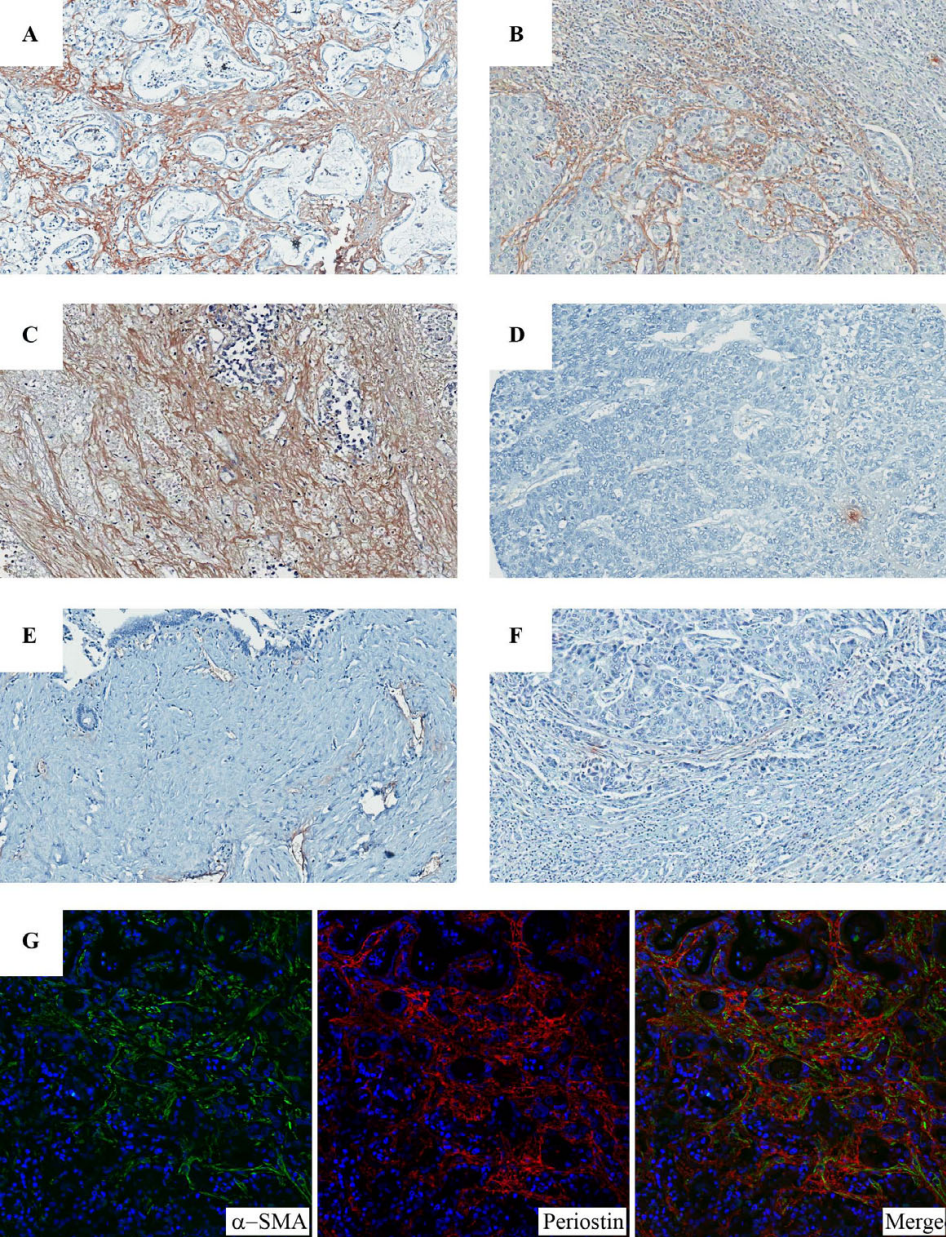
**Immunohistochemical staining of PN in CCA tissues**. The expression of PN was localized in fibroblasts but not cancer cells. High expression of PN was observed in well- (A), moderately- (B) and poorly-differentiated tissues (C), whereas PN negative staining CCA tissue was demonstrated (D). Benign liver tissue (E) and hepatocellular carcinoma (F) showed no to slight expression. Magnification, 100×. Double immunofluorescence staining showed co-expression of PN and α-SMA in CCA stromal fibroblasts (G). Magnification, 200×.

Immunohistochemistry revealed that the expression of PN was exclusively localized in fibroblasts but not cancer cells (Fig 3). Of all 52 cases, 43 cases or 83% were PN positive (Table 4). Among these positive cases, 58% of them showed high expression levels. High expression of PN was observed in well- (Fig 3A), moderately- (Fig 3B) and poorly-differentiated malignant tissues (Fig 3C). For PN-negative CCA tissues, only 17% (9/52) were in this group in which no PN was detected in either fibroblasts or cancer cells (Fig 3D). In contrast, benign liver tissues showed no (2/8) to slight (6/8) PN expression. Similar to benign liver tissues, hepatocellular carcinoma revealed low PN expression in their stromal cells (Fig 3E and 3F). Moreover, double immunofluorescence staining revealed co-localization of α-SMA and PN in the fibroblasts within cancerous area (Fig 3G).

**Table 4:** PN expression in CCA tissues compared to benign liver tissues and hepatocellular carcinoma.

Tissues	Total cases (n)	PN expression in fibroblasts		
		
		Negative	Positive	
			Low	High
CCA	52	9 (17%)	13 (25%)	30 (58%)
Benign liver tissue	8	2 (25%)	6 (75%)	0 (0%)
Hepatocellular carcinoma	4	1 (25%)	3 (75%)	0 (0%)

**Figure 4:**
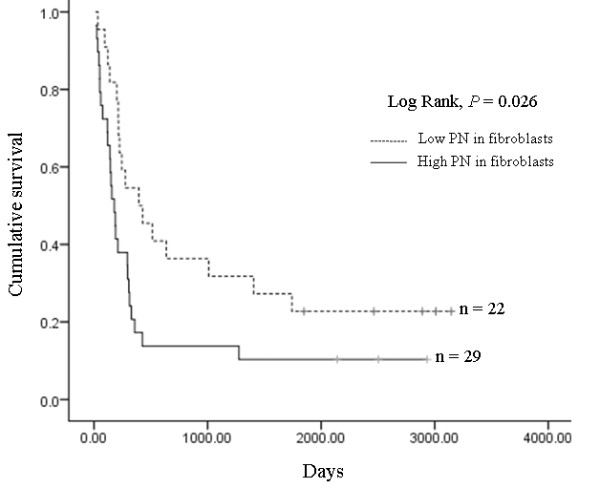
**Multivariate analysis using Kaplan-Meier method**. Cumulative survival analysis showed significantly shorter survival time of the patients with high PN expression in fibroblasts when compared to those who had low PN expression in fibroblasts (*P *= 0.026).

Cumulative survival of CCA patients with low or high PN expression in cancer stromal fibroblasts was analyzed using the Kaplan-Meier method. The patients with survival time under 14 d were identified as peri-operative deaths (n = 1) and excluded from the analysis. Median survival time was 395 ± 157 d for patients with low and 179 ± 35 d for patients with high PN expression. We found that the patients with high PN positive fibroblasts had statistically significantly shorter survival times than those with low PN positive fibroblasts (*P *= 0.026) (Fig 4). The prognostic value of PN expression and other clinicopathological factors among CCA patients was analyzed using multivariate Cox Proportional Hazard Regression model. The results revealed that high PN expression (HR = 2.02, *P *= 0.045), and the presence of lymph node metastasis (HR = 3.13, *P *= 0.002) were the independent risk factors for the overall survival of CCA patients after hepatectomy (Table 5). However, lymph node metastasis and other clinical data showed no association with PN expression (Table 6).

**Table 5:** Multivariate analysis by Cox proportional hazard regression model for the evaluation of prognostic factors.

Variable (No. of patients)	No. of dead patients	Hazard ratio	95% confidence interval	*P*
	(5-yr survival cut-off)	(HR)	(CI)	
**Age in years**				
≤ 57 (25)	21	1		
>57 (26)	22	1.25	0.62-2.48	0.533
**PN expression**				
Low (22)	17	1		
High (29)	26	2.02	1.02-4.02	0.045*
**Lymph node metastasis**				
Absence (36)	28	1		
Presence (15)	15	3.13	1.54-6.35	0.002*
**Histological type**				
Well-differentiated (20)	16	1		
Moderately-differentiated (8)	8	2.77	1.10-6.98	0.031*
Poorly-differentiated (8)	7	1.64	0.63-4.29	0.310
Papillary (15)	12	0.60	0.25-1.44	0.254
**Tumor size (cm)**				
≤ 5 (28)	23	1		
>5 (23)	20	1.49	0.76-2.94	0.251

* *P *value of equal or less than 0.05 means statistical significance

**Table 6:** Correlation between PN expression level and clinicopathological parameters.

Variable	n	PN expression (%)		Univariate analysis	Multivariate analysis	
		
		Low	High	*P*	HR	*P*
**Age in years**				0.575		
≤ 57	26	10 (38.5)	16 (61.5)		1	
>57	26	12 (46.2)	14 (53.8)		0.899	0.870
**Sex**				0.375		
Female	20	10 (50.0)	10 (50.0)		1	
Male	32	12 (37.5)	20 (62.5)		1.638	0.452
**Histological type**				0.083		
Well-differentiated	21	8 (38.1)	13 (61.9)		1	
Moderately-differentiated	8	1 (12.5)	7 (87.5)		3.720	0.271
Poorly-differentiated	8	6 (75.0)	2 (25.0)		0.184	0.86
Papillary	15	7 (46.7)	8 (53.3)		0.611	0.505
**Tumor size (cm)**				0.123		
≤ 5	29	15 (51.7)	14 (48.3)		1	
>5	23	7 (30.4)	16 (69.6)		2.493	0.161
**Lymph node metastasis**				0.830		
Absence	37	16 (43.2)	21 (56.8)		1	
Presence	15	6 (40.0)	9 (60.0)		1.459	0.590

### PN promotes proliferation and invasion of CCA cells

**Figure 5:**
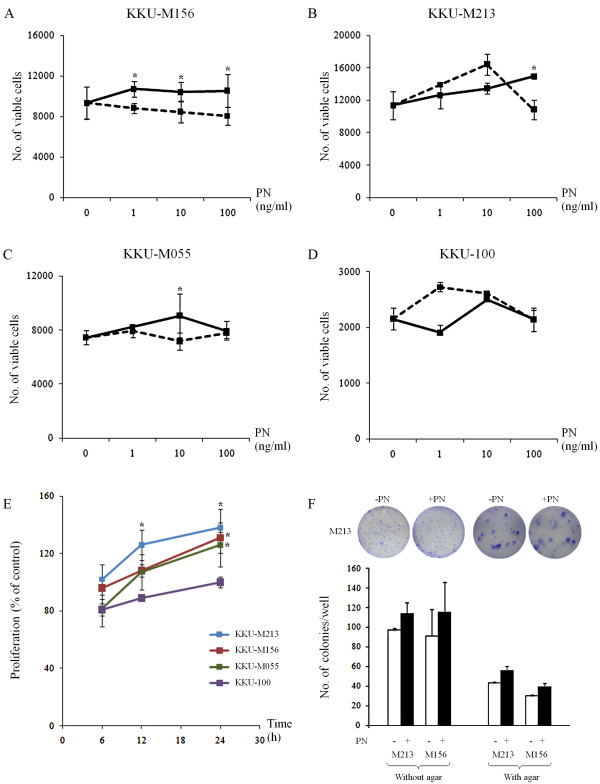
**PN promotes cell proliferation of CCA cell lines**. KKU-M156, KKU-M213 and KKU-M055 showed significantly induced proliferation with different concentrations of PN (A-C) whereas KKU-100 was unresponsive to PN (D). With an optimal dose of PN for each cell line, KKU-M213, KKU-M156, and KKU-M055 increased cell proliferation in a time-dependent manner (E). Triplicate experiments were performed for each assay. Results are expressed as mean ± SD and an asterix represents a *P *value less than 0.05 when compared to the negative controls without PN treatment. Black and dashed lines represent cells with and without PN treatment, respectively. Colony formation assay with and without agar was performed (F). Colony of more than 30 cells was counted under inverted microscope. Numbers of colonies/well in 6-well plate of both KKU-M213 and KKU-M156 CCA cell lines were higher in condition of PN treatment than those without PN. Results are expressed as mean ± SD of duplicate experiments. Pictures of crystal violet-stained cells are of KKU-M213 CCA cells in comparison between with and without PN.

**Figure 6:**
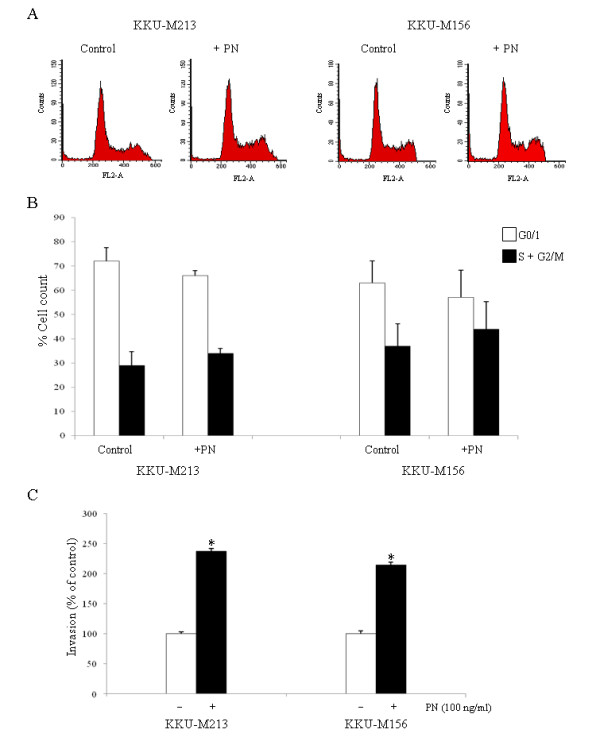
**Cell cycle distribution and invasion analysis of cancer cells with and without stimulation by PN**. Cell cycle analysis of KKU-M213 and KKU-M156 induced by PN (A). PN could drive cells from G1 into S and G2/M phases of the cell cycle when compared to control cells without PN treatment (B). Invasion induction by PN on KKU-M213 and KKU-M156 CCA cell lines is shown (C). Numbers of invaded cells when no PN was used served as control and were adjusted to be 100% (white bar). The increase of invaded cells induced by PN is observed and shown by a black bar. Each bar graph represents mean ± SD of three independent experiments. An asterix represents a *P *value of less than 0.05.

PN could induce proliferation of KKU-M156, KKU-M213 and KKU-M055 CCA cell lines (Fig 5A-C), but not KKU-100 (Fig 5D). In addition, KKU-M156, KKU-M213 and KKU-M055 responded to the proliferative effect of optimal PN concentration in a time dependent manner with statistical significance at the 24 h-treatment for all cell types (Fig 5E). To reinforce the proliferation effect of PN on CCA cell lines, colony formation assay with and without soft agar were performed and the result indicated the increased numbers of colonies in the condition of PN treatment in comparison to the negative control without PN stimulation (Fig 5F). In addition, flow cytometric analysis indicated an increased number of KKU-M213 and KKU-M156 cells distributed in S+G2/M when exposed to PN (Fig 6A and 6B).

To address the invasion effect of PN on CCA cells, the invasion assay of cell lines with high *ITGα*_5 _expression was performed in a Boyden chamber. The results showed that exogenous PN could markedly induce invasion of KKU-M156 and KKU-M213 CCA cell lines up to around 210% and 230% of cells without PN treatment (Fig 6C).

### Knockdown of ITGα5 attenuates PN-induced proliferation and invasion

**Figure 7:**
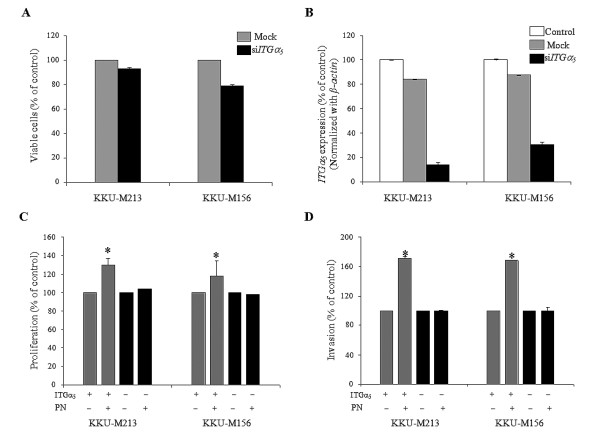
**Effect of si*ITGα*_5 _on PN-induced proliferation and invasion of CCA cells**. Lipofectamine-treated cells (mock) had nearly the same viability as si*ITGα*_5_-treated cells (A). KKU-M213 and KKU-M156 showed dramatically decreased expression of *ITGα*_5 _detected by real time RT-PCR after exposure to si*ITGα*_5 _(B)_. _Percentage of proliferation and invasion compared to control mock cells is shown. The si*ITGα*_5_-treated cells (negative ITGα_5_) could not respond to PN in induction of proliferation as much as that detected in cells without exposure to si*ITGα*_5 _(positive ITGα_5_) (C). A similar effect was observed in PN-induced invasion of CCA cell lines (D). Results represent mean ± SD of three independent experiments and an asterix represents significantly increased cell proliferation and invasion by PN compared to mock cells without PN treatment.

Treatment of CCA cells with si*ITGα*_5 _and lipofectamine (mock) for 6 h did not affect cell viability (Fig 7A). The reduction of *ITGα*_5 _expression was observed to be 88% of that expressed in both KKU-M213 and KKU-M156 CCA cells without transient knockdown of this gene (Fig 7B). The knockdown effect could be detected up to 72 h after si*ITGα*_5 _treatment (data not shown). Thus, the subsequent investigations of cell proliferation and invasion were done within 72 h after transient knockdown with si*ITGα*_5_.

The reduction of *ITGα*_5 _expressions in both KKU-M213 and KKU-M156 CCA cells resulted in a significant decreased response of cells to PN-induced cell proliferation and invasion (Fig 7C and 7D). A 104% of KKU-M213 cell proliferation induction was detected in si*ITGα*_5_-treated cells exposed to PN, whereas cell proliferation could increase up to 130% in cells with intrinsic *ITGα*_5_ expression (Fig 7C). In the same manner, KKU-M156 showed 118% and 98% of cell proliferation induction observed in cells untreated and treated with si*ITGα*_5_. Both *ITGα*_5_-knockdown CCA cell lines did not respond to PN-activated cell invasion whereas PN dramatically induced invasion of both cell lines having normal intrinsic *ITGα*_5 _expression (168% for KKU-M156 and 172% for KKU-M213) (Fig 7D). Finally, cells with high *ITGα*_5 _expressions were more susceptible to PN stimulation to proliferate and invade than cells without or minimal *ITGα*_5 _expression.

## Discussion

Cancer-associated fibroblasts have been recognized for their impact in the genesis, promotion and progression of many carcinomas and highlighted in several reviews [8,25]. CCA is notoriously associated with dense desmoplastic stroma with activated fibroblasts [5,6]. Relatively little, however, is known about the contribution of the stromal fibroblasts to CCA. The authors in the present group have recently shown that CCA stromal fibroblasts, with and without direct interaction with cancer cells could induce cancer cell proliferation [6]. Herein, this study focused on the gene expression profile of CCA-derived fibroblasts in order to investigate the molecular mechanism of how fibroblasts induce a favorable microenvironment to promote cancer. Even though the current study is limited to a single cancer fibroblast line isolated from a single CCA patient, the validity of array results was strengthened by comparing gene expression levels in cancer fibroblasts to the two lines of normal fibroblasts; one isolated from the same CCA patient and the other from a second patient. Only genes in cancer fibroblasts altered from both normal fibroblast lines were investigated as the common up- or down-regulated genes. This is to provide evidence that the fibroblasts used in our study are valid representatives of fibroblasts found in CCA.

By comparing gene profiles in fibroblasts from CCA with those of other tumor types, it is suggested that CCA fibroblasts display not only common genotypes for activated cells but also unique characteristics. Genes involved in metabolism of cells needed to be up-regulated in order to support the active function of CCA stromal fibroblasts to produce many supporting proteins in the cancer environment. Neuropeptide Y receptor Y1 has been indicated to receive the activation signal to induce neuroproliferation [26] and doublecortin-like kinase 1, a microtubule-associated active protein kinase expressed in growth cones of postmitotic neurons [27] may help facilitate fibroblast proliferation. In similar to human basal cell carcinoma fibroblasts [14], *SPARC *or osteonectin, was also over-expressed in CCA-derived fibroblasts. *SPARC*-null mice were recently demonstrated to resist UV-induced squamous cell carcinoma, suggesting a tumor-promoting role of *SPARC *[28]. In contrast to the cancer-associated fibroblasts in metastatic colon cancer to the liver which showed down-regulation of *SDF-1 *[18], CCA-derived fibroblasts had up-regulated *SDF-1 *(data not shown).

The highly up-regulated genes in CCA-derived fibroblasts showed several interesting functions involved in cancer progression. Serpin peptidase inhibitor, clade B member 2 (*SERPINE2*) or plasminogen activator inhibitor type 2 (*PAI2*) is involved in cancer invasion and metastasis by controlling serine protease urokinase plasminogen activator. In a recent review, several studies led to the suggestion that the significance of *PAI2 *expression on prognosis of cancers is organ context-dependent [29]. In breast cancer, *PAI2 *was expressed in both stromal and tumor cells and associated with prolonged disease-free survival [30]. In contrast, high levels of *PAI2 *in endometrial cancer were reported to correlate with the invasion potential of the cancer [31]. S100 calcium binding protein A4 (*S100A4*) has been revealed as the metastasis-inducing protein [32]. Genes such as procollagen C-endopeptidase enhancer 2 (*PCPE2*) were also detected which may involve in collagen synthesis [33]. These results support the function of fibroblasts in CCA to promote a desmoplastic reaction. For down-regulated genes, bone morphogenetic protein 2 (*BMP2A*), a multi-functional growth factor belonging to the transforming growth factor-β superfamily was decreased in CCA fibroblasts as reported in breast cancer-derived fibroblasts [13]. *BMP2A*-encoding protein has been elucidated to induce hypophosphorylation of retinoblastoma protein causing cell cycle arrest [34]. Hence, decreased BMP2A in the CCA microenvironment may promote cancer cells to enter the cell cycle. Moreover, a decreased level of interleukin 24 (*IL-24*), an apoptotic inducible cytokine [35], in cancer tissues, attenuates cancer cells from undergoing apoptosis. The response gene to complement 32 (*RGC32*), a novel p53-inducible gene, and bradykinin receptor B1 (*BRADYB1*) decreased expression in CCA-derived fibroblasts. Being intracellular protein and membrane receptor, respectively, *RGC32 *and *BRADYB1 *have elucidated the function of inhibition of fibroblast cell proliferation [36,37]. It seems possible to conclude that down-regulated genes in fibroblasts encode proteins, if acting in the intracellular region, can inhibit the proliferation of fibroblasts themselves, but if they exist in the extracellular region, they may involve inhibition of cancer cell proliferation. This evidence strengthens the roles of fibroblast-derived proteins released into a tumor environment to induce a high proliferative capability of cancer cells.

Fibroblasts have been proposed the bipolar effects in cancers [38]. In our microarray results, *ADAMTS-like 1 *(*ADAMTSR1*) was over-expressed in CCA-derived fibroblasts. The ADAMTS-like proteins have been discussed as the enhancers of ADAMTS proteases [39]. Since some ADAMTS have been proven to be anti-angiogenic factors [40] partly via the trapping of vascular endothelial growth factor by thrombospondin motifs of ADAMTS [41]. So up-regulation of *ADAMTSR1 *in fibroblasts may inhibit angiogenesis. Moreover, *stromelysin-1 *or *MMP-3 *which can degrade ECM and induce cancer invasion and metastasis, showed the decreased expression in fibroblasts. Taken together, the increased expression of *ADAMTSR1 *and the decreased expression of *MMP-3 *may highlight fibroblasts in term of suppressing CCA progression.

Theoretically, proteins secreted from fibroblasts having interplay with cancer cells could be detected in the extracellular region and be involved in ECM organization and biosynthesis. Within these 2 groups of genes, we focused our interest on genes encoded secreted proteins and their products have been previously reported of their tumorigenic effects. *ADAM12*, *AREG*, *AGN2, ER, JAGL1, LAMA5, NOV*, *PDGF-A*, *PN*, *RL*, and *SCG2 *were selected to explore. *AREG*, *ER*, *JAGL1*, and *LAMA5 *are predominantly reported for proliferation induction in cancer cells [42-45]. *PDGF-A*, *NOV*, *AGN2*, and *SCG2 *are involved in angiogenesis [46-49], whereas *ADAM12 *and *RL *play an important role in cell motility, invasion and metastasis [50,51]. For *PN*, many carcinogenic functions including cell proliferation, invasion, metastasis and angiogenesis have been demonstrated [20-24]. This study employed real time PCR to verify the up-regulation of these genes, and found that only *ADAM12*, *AREG*, *ER*, *JAGL1*, *PDGF-A*, *PN *and *SCG2 *were significantly increased in their expression levels in CCA-derived fibroblasts and may promote CCA progression through activation of cancer growth, invasion and angiogenesis.

Herein PN was chosen to deeply explore since its well accepted multifunction in cancer as mentioned above. Moreover, the result from our group about the expressions of *ADAM12*, *AREG*, *ER*, *JAGL1*, *PDGF-A*, *PN *and *SCG2 *in whole CCA tissues (n = 20) showed that only *AREG*, *PDGF-A *and *PN *had higher level in cancer than those in benign liver tissues with statistical significance (data not shown). *AREG *and *PDGF-A *could be detected in not only fibroblasts but also in cancer and endothelial cells, however *PN *expressed exclusively in CCA fibroblasts. In order to demonstrate role of fibroblast-derived proteins in CCA, we determined that *PN *should be the first target to explore.

The strong evidence using different biological preparations of CCA-derived fibroblasts and CCA tissues confirmed the increased levels of PN at both mRNA and protein. Most of CCA tissues of all differentiated types had high levels of PN and expressed exclusively in α-SMA positive fibroblasts. In the same direction, the findings showed no expression of PN in CCA cell lines when compared to the high level expressed in the fibroblasts. This may strengthen the results of the absence of PN in cancer cells in CCA tissues. From these results taken together, it can be concluded that PN detected in CCA tissues is only of fibroblast origin as reported in some cancers [52-54]. In cancers of head and neck, ovary, and colon, PN was found in cancer cells and has been proposed to induce tumorigenic properties of cancer cells via an autocrine mechanism [21,22]. Hence results from the present study allow the speculation to propose a phenomenon that fibroblast-derived PN in CCA may affect cancer cells by a paracrine mode and has a promising role in cancer promotion. These results revealed that a high PN level in fibroblasts was an independent risk factor in CCA patients and those having high PN had significantly low cumulative survival time after surgery. PN might therefore be used as a poor prognostic marker in patients suffering from CCA. Detections of PN at both mRNA and encoding protein in CCA tissues are in the same direction to distinguish CCA from non-cancer syndromes of bile ducts. In addition, most benign liver tissues and hepatocellular carcinoma showed no to only a minimal expression of PN when compared to the high level detected in CCA tissues. Hence, serum PN may help to distinguish CCA from benign conditions and closely-related liver cancer and may use as the prognostic or predictive marker as previously reported [52,55].

To show the tumorigenic impacts of PN on CCA cells, recombinant PN was employed as extracellular PN to mimic the paracrine effect of PN produced from cancer stromal fibroblasts to induce CCA cell proliferation and invasion. Though receptors ITGα_v_β_3 _and ITGα_v_β_5 _have been shown to be the receptors for PN in several cancer cells [21], PN promoted invasiveness of pancreatic cancer cells via the β_4 _integrin [56]. This suggests the cell type dependent on a specific ITG responded to PN. The study herein reveals that PN-induced cell proliferation and invasion could be inhibited by RNAi against *ITGα*_5. _Hence, ITGα_5 _is a potentially promising receptor for PN in CCA cells. As the well known receptor for fibronectin, the apparent reason for ITGα_5 _production in CCA cells is to support the abundance of fibronectin found in CCA [57]. In addition, ITGα_5 _can only be from dimerization with the β_1 _subunit and activation of ITGα_5_β_1 _has been revealed to support cell survival [58] and induce invasion and angiogenesis [59,60]. Though further studies need to be performed before such a conclusion is valid in CCA, this work highlights the PN-induced-ITGα_5 _pathway as one of the activated pathways to induce an aggressive CCA.

TGF-β has been proposed to induce the expression of *PN *[61]. *O. viverrini *excretory/secretory product has also recently been shown to be the stimulator of fibroblast proliferation via the TGF-β-mediated signal transduction pathway [62] and this pathway seems likely to be the cause of *PN *expression in CCA-derived fibroblasts. The authors' laboratory has checked the effect of parasitic product-treated fibroblasts and found that these fibroblasts increased *PN *expression compared to the normal liver fibroblasts without exposure (unpublished data). It is interesting to propose that in CCA cells; the expression of fibroblast-derived PN could be induced by TGF-β produced from infected parasites since the early stage of carcinogenesis and may be in concert with TGF-β produced from CCA cells in a late stage of cancer [12]. Though *in vivo *experiments are needed to confirm, fibroblast-derived PN may influence *O. viverrini*-associated CCA at the early stage of cancer as well as to promote cancer progression in the later time. With this information, targeting the stroma in CCA may not only be effective in treatment of primary, invasive and metastatic tumors, but may also play role in prevention of tumor development.

## Conclusions

**Figure 8:**
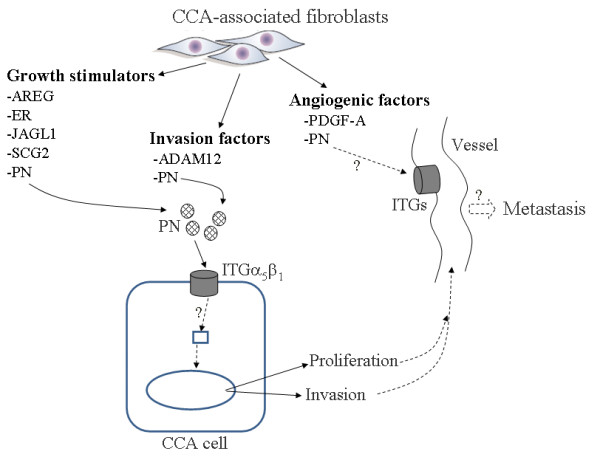
**Proposed impacts of CCA-associated fibroblasts revealed in this study**. A schematic representation of the main alterations in CCA-associated fibroblasts revealed in this study. The biological functions of protein products from the up-regulated genes in fibroblasts are represented. Tumorigenic effects of PN on CCA cancer cells are also proposed.

To the authors' knowledge, this study is the first to describe the gene expression profile of CCA-derived fibroblasts. Molecular understanding of fibroblasts in CCA by the functions of certain up- and down-regulated genes has been revealed and has suggested certain groups of genes in controlling cancer cell proliferation, invasion, metastasis and angiogenesis (Fig 8). These findings provide evidence that fibroblasts are important sources of tumorigenic substances, particularly PN, when produced into the microenvironment of CCA. High levels of PN are found in most CCA patients and can be used as a poor prognostic marker. In addition, the level of PN can be used to distinguish CCA from other benign liver conditions and hepatocellular carcinoma. The interaction of fibroblast-derived PN and CCA cells helps to promote cell proliferation and invasion probably via ITGα_5_. Though further investigations are needed, this study suggests promising evidence of the value of using serum PN as a prognostic marker of poor survival in CCA patients. Moreover, targeting fibroblasts or fibroblast-derived-PN-stimulated pathways in cancer cells to attenuate the tumorigenic induction of PN is a further challenge to inhibit CCA progression in the patients.

## Methods

### Cells and culture condition

Cfs and Lfs were established and characterized in this lab as previously reported [6]. CCA cell lines including KKU-M213; KKU-M156; KKU-M055; and KKU-100 were received as a kind gift from Associate Professor Dr. Banchob Sripa (Khon Kaen University). Cfs and Lfs were grown in the complete media which is 10% FBS containing DMEM with 20% epidermal growth factor (CytoLab Ltd., Rehovot, Israel). CCA cells were cultured in 10% FBS containing Ham-F12 (Invitrogen, Carlsbad, CA) supplemented with antibiotics and an anti-fungal agent at 37°C and in 5% CO_2 _incubator.

### Gene expression profiling study and data analysis

Total RNA was extracted using RNeasy Micro Kit (Qiagen, Valencia, CA) using the manufacturer's instructions. The quality of the RNA was assessed by an Agilent RNA 6000 Nano Kit (Agilent Technologies, Waldbronn, Germany). Affymetrix GeneChip Human Genome U133 plus 2.0 Array containing 38,500 human genes (Affymetrix, Santa Clara, CA) was used following Affymetrix's instruction. The array data were scanned by a GeneChip Scanner 3000 (Affymetrix) and analysed by Affymetrix microarray suite, version 5.0. Raw data from the GeneChips were used to analyze expression levels and expressed as fold changes and gene ontology was categorized by Gene Spring G.X.7.3 software (Agilent Technologies). Average fold change of gene expression was determined by intensity comparison between Cf and Lf1 and Lf2.

### Semi-quantitative real time PCR

Complementary DNA was synthesized from 1 μg of total RNA using the first strand cDNA synthesis kit (AMV) (Roche Molecular Biochemicals, Mannheim, Germany) according to the instructions. Relative expression levels in genes of Cfs and Lfs were determined by SYBR Green-based real time PCR using ABI 7500 (Applied Biosystem, Foster City, CA) and calculated by the 2^-ΔΔC^_T _equation. In this case, Δ*C*_T _= C_T _(Cf)-C_T _(Lf). *β-actin *served as an internal control to adjust the amount of starting cDNA. The sequences of genes tested in this study were retrieved from PubMed http://www.ncbi.nlm.nih.gov and the primers were designed by Primer 3 (Table 7).

**Table 7:** Primer sequences for real time PCR.

Gene	Forward Primer	Reverse Primer	Size	**Accession no**.
	5'-3'	5'-3'	(bp)	
*ADAM12*	tttgggggtcaacagttttc	agagctgggttcccttttgt	191	NM_003474
*AREG*	tggggaaaagtccatgaaaa	tttcgttcctcagcttctcc	174	NM_001657
*AGN2*	ccacctgaggaactgtctcg	ggtcttgctttggtccgtta	191	NM_001147
*ER*	catatgggagaagggggagt	aagtgcaattacagagtgcaaaa	166	NM_001432
*JAGL1*	gcctgccttaagtgaggaaa	gccaagaacaacacatcaaaga	169	U77914
*LAMA5*	gtgatgaaaagcgggaatgt	acctccacagagcgagtcat	221	BC003355
*NOV*	tgcaattccaagaaaatatcactg	cttggatttggagcttggaa	167	NM_002514
*PDGF-A*	acacgagcagtgtcaagtgc	tctggttggctgctttaggt	250	X03795
*PN*	cactctttgctcccaccaat	tcaaagactgctcctcccata	157	AY140646
*RL*	tgctgaatttggggctactt	gggagatagggtcttcatcca	198	NM_005045
*SCG2*	cccgaagaatgatgataccc	aaatgttgggatttgcttgg	195	NM_003469
*ITGα*_5_	agttgcatttccgagtctgg	ccaaacaggatggctaggat	223	NM_002205
*β-actin*	cacactgtgcccatctacga	ctccttaatgtcacgcacga	162	X00351
*gapdh*	ctcctcctgttcgacagtca	gttaaaagcagccctggtga	140	NM_002046

Note: *ADAM12*, a disintegrin and matrix metalloproteinase 12; *AREG*, amphiregulin; *AGN2*, angiopoietin 2; *ER*, epiregulin; *JAGL1*, jagged soluble form; *LAMA5*, laminin alpha 5; *NOV*, nephroblastoma over expressed; *PDGF-A*, platelet-derived growth factor alpha; *PN*, periostin; *RL*, reelin; *SCG2*, secretogranin 2; *ITGα5*, integrin alpha 5; *β-actin*, beta-actin; *gapdh*, glyceraldehyde 3-phosphate dehydrogenase

### Human CCA tissues and immunohistochemistry

Fifty-two cases of CCA tissues were obtained from patients who had undergone hepatectomy using the protocol approved by the Human Research Ethics Committee, Khon Kaen University (HE490143). The age, sex, tumor size, histological type and staging data were derived from the medical charts and pathological records. Benign liver tissues were characterized as chronic inflammation by other causes rather than CCA.

Paraffin-embedded tissues were used and antigens were retrieved in 10 mM citrate buffer pH 6.0 at 95°C for 40 min and endogenous peroxidase was blocked in 3% H_2_O_2 _for 5 min. After blocking non-specific binding with 2% bovine serum albumin for 20 min, 1:10,000 rabbit anti-human PN (Biovendor, Heidelberg, Germany) was applied to the sections at room temperature overnight, followed by anti-rabbit Envision^+ ^System-HRP labeled polymer (Dako, Carpinteria, CA) for 30 min at room temperature. The immunoreactive signal was developed by diaminobenzidine (DAB; Sigma, St Louis, MO) and counterstained with hematoxylin. The signal was checked under light microscope.

PN expression of intratumoral fibroblasts on the histologic sections was semi-quantitatively scored on the basis of PN-positive fibroblasts percentage and the immunostaining intensity. The number of positive fibroblast cells were classified as < 10% (negative); 10-25% (+1); 26-50% (+2); and >50% (+3). The intensity of PN expression in fibroblasts was scored no staining, 0; weak staining, 1; intermediate or focal weak and focal intense staining, 2; intense staining, 3. The interpretation of PN expression was performed by summarization the scores of the percent positive cell (0-3) and the scores of staining intensity (1-3) to reach the total final score of 0-6. The results were then categorized as follows; low expression, score ≤ 4; and high expression, score > 4. All samples were anonymized and independently scored by one pathologist (KC) and 2 investigators (PT and CT). In case of disagreement, the slides were reexamined and a consensus was reached by at least 2 observers.

### Double immunofluorescence staining of α-SMA and PN

In order to localize the expression of PN and α-SMA in CCA tissues, double immunofluorescence staining was performed. The 1:200 mouse anti-human α-SMA antibody (Sigma) and 1:500 rabbit anti-human PN antibody (Biovendor) were used as primary antibodies. Anti-mouse IgG-Alexa 488 and anti-rabbit IgG-Cy3 (Invitrogen) were used as the second antibodies. Nucleus was stained with Hoechst (Invitrogen). The signal was observed under the LSM 510 Meta laser scanning confocal microscope (Carl Zeiss, Jena, Germany) at the Division of Medical Molecular Biology, Office for Research and Development, Faculty of Medicine Siriraj Hospital, Mahidol University.

### Protein extraction and western blot analysis

Twenty μgs of total protein from the cell lysate were separated in 10% SDS-PAGE and transferred onto a PVDF membrane (Millipore, Billerica, MA). For PN detection, 1:500 rabbit anti-human PN (Biovendor) and 1:1,000 goat anti-rabbit conjugated HRP (Abcam, Cambridge, MA) were used. The signal was visualized by ECL (Pierce, Rockford, IL). The expression of β-actin was used as an internal control to determine an equal amount of loading proteins.

### Cell proliferation assay

CCA cells with or without treatment with si*ITGα*_5 _cells were arrested in HAM-F12 without serum supplement for 12 h. Different concentrations of recombinant PN (Biovendor) prepared in 1% FBS containing HAM-F12 were incubated with cells for 6, 12 and 24 h. The viable cells in each condition were determined using an MTS assay (Promega, Madison, WI) according to the manufacturer's instruction.

### Cell cycle analysis by flow cytometry

Cell cycle distribution analysis used cells stained with propidium iodide (Invitrogen) as previously described [63]. The distribution of cells in each stage of the cell cycle was quantitated in a flow cytometer and CellQuest software (Becton Dickinson, Franklin Lakes, NJ). Numbers of CCA cell lines in S+G2/M phases of the cell cycle were measured and compared between conditions with and without recombinant PN treatment. These experiments were repeated two times using replicate culture dishes in the same experiment.

### Colony formation assay

CCA cell lines were cultured in 6-well plate. After 24-h culture, recombinant PN diluted in 1% FBS containing medium was added and the plate was incubated in CO_2 _incubator. Soft agar colony formation assay was also performed using 0.5% and 0.35% MetaPhor^® ^agarose (Cambrex Bio Science, Rockland, ME) as lower and upper layers, respectively. After 12 d, cells were fixed with 5% v/v glutaraldehyde and stained with 0.5% w/v crystal violet in 40% v/v methanol. Cell growth was estimated by counting numbers of colonies with more than 30 cells under inverted microscope and compared between those of treat and untreated with PN. The experiment was performed in duplicate.

### Invasion assay

KKU-M213 and KKU-M156 CCA cells were seeded in PN (100 ng/ml) containing medium into the Matrigel invasion chamber (BD Biosciences, San Jose, CA) and incubated for 24 h. Invaded cells were fixed with 5% v/v glutaraldehyde and stained with 0.5% w/v crystal violet in 40% v/v methanol for 30 min each. The number of invaded cells was counted under a microscope by two independent investigators using 100× magnification fields. The assays were done in replicate and three independent experiments were performed.

### Small interfering RNA against receptor integrin α_5_

Two hundred thousand CCA cells were seeded into a 6-well plate for 24 h before transfection of si*ITGα*_5 _(Santa Cruz Biotechnology, Santa Cruz, CA) by Lipofectamine 2000 (Invitrogen). Three siRNA strands (5'-gucagaauuucgagacaaa-3', 5'-caccaacaagagagccaaa-3', and 5'-ccacugaccagaacuagaa-3') were used to target *ITGα*_5 _mRNA. The efficiency of knock down was tested by real time PCR using *β-actin *as an internal control.

### Statistical analysis

Statistical analyses were performed using SPSS version 16.0 (SPSS Inc., Chicago, IL). The correlation of PN expression and pathological parameters of CCA patients was analyzed by the χ^2^-test and binary logistic multivariate analysis. Patient survival was calculated from the time of surgical resection to death and the survival curves were constructed according to Kaplan-Meier, with a Log-Rank test. A multivariate analysis was performed by the Cox proportional hazard regression model. The significance of the different data was determined by the Student's t-test. A *P *value of equal to or less than 0.05 was defined as statistically significant.

## Competing interests

The authors declare that they have no competing interests.

## Authors' contributions

KU performed most of the experiments and helped to draft the manuscript. YA contributed to the microarray experiment. PT performed real time PCR of some genes and helped KC and AP in immunohistochemical scoring. SC contributed to the patient clinicopathological data and samples collection. CT contributed to the design of the entire study, data analysis and preparation of the manuscript.

All authors have read and approved the final manuscript.
